# Effect of high-frequency loading and parathyroid hormone administration on peri-implant bone healing and osseointegration

**DOI:** 10.1038/s41368-018-0009-y

**Published:** 2018-03-13

**Authors:** Aya Shibamoto, Toru Ogawa, Joke Duyck, Katleen Vandamme, Ignace Naert, Keiichi Sasaki

**Affiliations:** 1 Division of Advanced Prosthetic Dentistry, Tohoku University Graduate School of Dentistry, Sendai, Japan; 2 Department of Oral Health Sciences, Prosthetic Dentistry, BIOMAT—Biomaterials, Katholieke Universiteit Leuven, Leuven, Belgium

## Abstract

The objective of this study is to examine the effect of low-magnitude, high-frequency (LMHF) loading, and anti-osteoporosis medications such as parathyroid hormone (PTH) and bisphosphonates on peri-implant bone healing in an osteoporosis model, and to assess their combined effects on these processes. Thirteen-week-old ovariectomized rats (*n* = 44) were divided into three groups: PTH, alendronate, and saline. After 3 weeks of drug administration, titanium implants were inserted into the tibiae. Each group was subdivided into two groups: with or without LMHF loading via whole-body vibration (50 Hz at 0.5 *g*, 15 min per day, 5 days per week). Rats were killed 4 weeks following implantation. Removal torque test, micro-CT analyses (relative gray (RG) value, water = 0, and implant = 100), and histomorphometric analyses (bone-to-implant contact (BIC) and peri-implant bone formation (bone volume/tissue volume (BV/TV))) were performed. Removal torque values and BIC were significantly differed by loading and drug administration (ANOVA). Post hoc analysis showed that PTH-treated groups were significantly higher than the other drug-treated groups. BV/TV was significantly enhanced by PTH administration. In cortical bone, RG values were significantly increased by loading. In trabecular bone, however, RG values were significantly increased by PTH administration. These findings suggest that LMHF loading and PTH can act locally and additively on the bone healing process, improving the condition of implant osseointegration.

## Implants: Hormone and vibration stimulus aid bone healing

Whole-body vibration and administration of a hormone used to treat osteoporosis can enhance bone healing at the site of a titanium implant. Toru Ogawa of Tohoku University Graduate School of Dentistry in Sendai, Japan, and colleagues gave anti-osteoporosis medications, either parathyroid hormone or the bisphosphonate drug alendronate, to female rat models of osteoporosis. After three weeks of drug administration or a saline control, the researchers inserted titanium implants into the rats’ leg bones. Half the rats were then exposed to whole-body vibration, which applies low-magnitude, high-frequency mechanical forces. A multitude of tests showed that parathyroid hormone improved bone healing at the implant more than alendronate or saline did. The vibrational stimulus further increased the healing. The findings suggest that these treatments could aid in oral bone healing for patients receiving dental implants.

## Introduction

Increasing human life expectancy has meant that greater consideration needs to be given to oral implantation in patients with systemic diseases. Osteoporosis is known as one of the systemic risk factors for implant failure,^1,2^ if without proper management, poor bone quality can result in the lack of primary stability, and impaired bone formation and healing can make achieving osseointegration difficult.

In the field of orthopedics and oral implantology, it is well known that bone adjust its mass and microstructure responding to mechanical loading. Low-magnitude, high-frequency (LMHF) loading, in which a low-magnitude is generally meant as <1 *g* (1 *g = *9.98 m·s^-2^) and a high-frequency is generally meant as 20–90 Hz, elicits a positive effect on the skeleton.^3,4^ Numerous studies suggest that LMHF loading, applied by means of whole-body vibration (WBV), stimulates bone formation and fracture healing.^5–8^ WBV loading has been used clinically as a nonpharmacological intervention in the treatment of osteoporosis.^9–14^

With regard to titanium implant osseointegration, which has similarities to bone fracture healing, previous studies have suggested that LMHF loading has an osteogenic effect on peri-implant bone.^15–17^ In particular, Ogawa et al.^15,18^ confirmed that the specific parameters of a loading regimen, such as duration, session distribution, frequency, and amplitude of loading, have important roles in the impact of LMHF loading on peri-implant bone. In addition, LMHF loading reportedly increased bone–implant osseointegration in osteoporosis models.^19–21^ However, the combined effect of LMHF loading and anti-osteoporosis medications, which are the most common treatment of osteoporosis, on peri-implant bone healing and implant osseointegration remains unclear.

Human parathyroid hormone (1-34) [hPTH(1-34)] is a new class of skeletal anabolic agents that stimulates osteoblastic bone formation and has already been approved for the treatment of osteoporotic patients who are at high risk for fractures or who have been intolerant of previous osteoporosis therapy. Intermittent systemic administration of PTH reportedly reduced the risk for fractures by improving bone microarchitecture and enhancing overall bone mass,^22–25^ and exceeded bisphosphonates (BP), which are important antiresorptive agents, in increasing bone mineral density.^26^ Therefore, PTH holds promise as an alternative to existing antiresorptive agents.^27^ There has been growing interest in the use of PTH to accelerate fracture healing and increase bone formation in the early post-operative period after osteosynthesis and following joint replacement with orthopedic implants.^28,29^ The effects of using PTH for maxillofacial bone formation and regeneration have also been investigated in recent years.^30^ Intermittent PTH administration reportedly increased osseointegration of titanium oral implants in animal and clinical models.^31–33^

BP, on the other hand, are antiresorptive agents that reduce osteoclastic bone loss and are widely used for prevention and as the first-choice therapy for osteoporosis. BP administration suppress bone resorption and bone remodeling, resulting in a relative increase of bone formation and bone mineral density and subsequently lowing the fracture rate.^34^ The current animal studies have reported its efficacy on implant osseointegration under osteoporotic conditions.^35^

Although both PTH and BP administration are effective for the management of osteoporotic condition, they have some limitations and side effects associated with the prolonged administration,^36–38^ which are possible complications of oral implantation.　Therefore, to treat osteoporotic patients with oral implants safely and successfully, it is necessary to reduce bone healing period and achieve early and strong osseointegration after implant surgery. LMHF loading may be a good consideration for a novel therapeutic option for efficient oral implantation in osteoporotic patients.

This study evaluated the hypothesis that LMHF loading and anti-osteoporosis medications have a beneficial effect on peri-implant bone healing and implant osseointegration in osteoporosis model. More specifically, the aim of this study was to investigate the impact of LMHF loading on peri-implant bone response for ovariectomized (OVX) rats treated with PTH versus BP, to assess which combination is more effective in enhancing implant osseointegration.

## Results

### Removal torque test

**Fig. 1:**
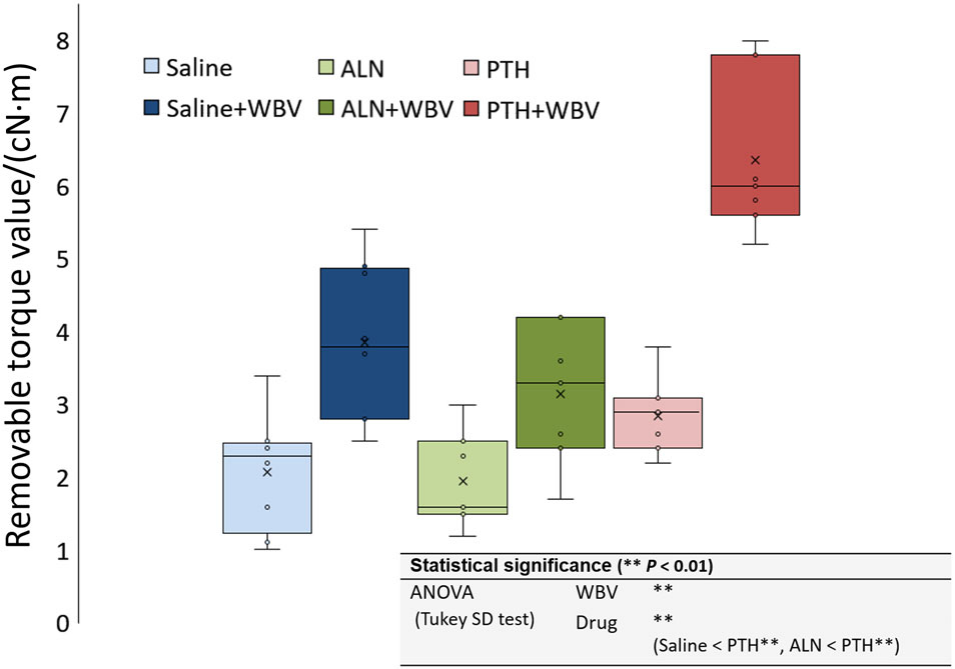
Results of removal torque (RT) test. The box-plot of the peak loosening torque (RT value), which was measured with a torque gauge. (**P < 0.01; two-way ANOVA followed by Tukey’s HSD test).PTH, parathyroid hormone; WBV, whole-body vibration; ALN, alendronate

RT values were significantly differentiated by the loading and the drug administration (*P* < 0.01) (Fig. 1). PTH administration significantly increased the biomechanical strength of the implant–bone interface compared with the saline-treated and alendronate (ALN)-treated groups (*P* < 0.01).

### Micro-CT analysis

**Fig. 2:**
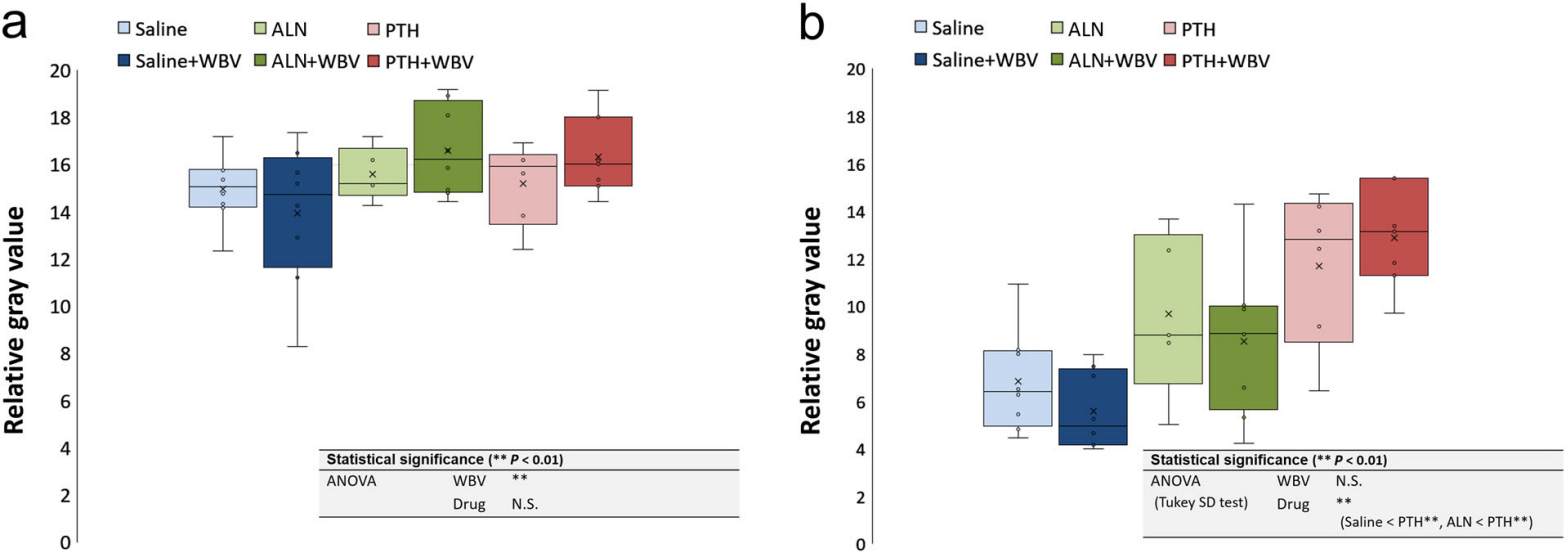
Results of micro-CT analysis. In **a** cortical and **b** trabecular bone. The box-plot of the relative gray (RG) value (water = 0 and titanium implant = 100) of each region of interest (ROI) (**P < 0.01; two-way ANOVA followed by Tukey’s HSD test). PTH, parathyroid hormone; WBV,whole-body vibration; ALN, alendronate

In the cortical bone surrounding the implant, RG values were significantly affected by the loading (*P* < 0.01) (Fig. 2a). There were no significant differences according to the type of drug. In trabecular bone, RG values of the PTH-treated groups were significantly higher than those of the saline-treated and ALN-treated groups (*P* < 0.01) (Fig. 2b). There were no significant differences according to the loading.

### Histologic and histomorphometric analysis

**Fig. 3:**
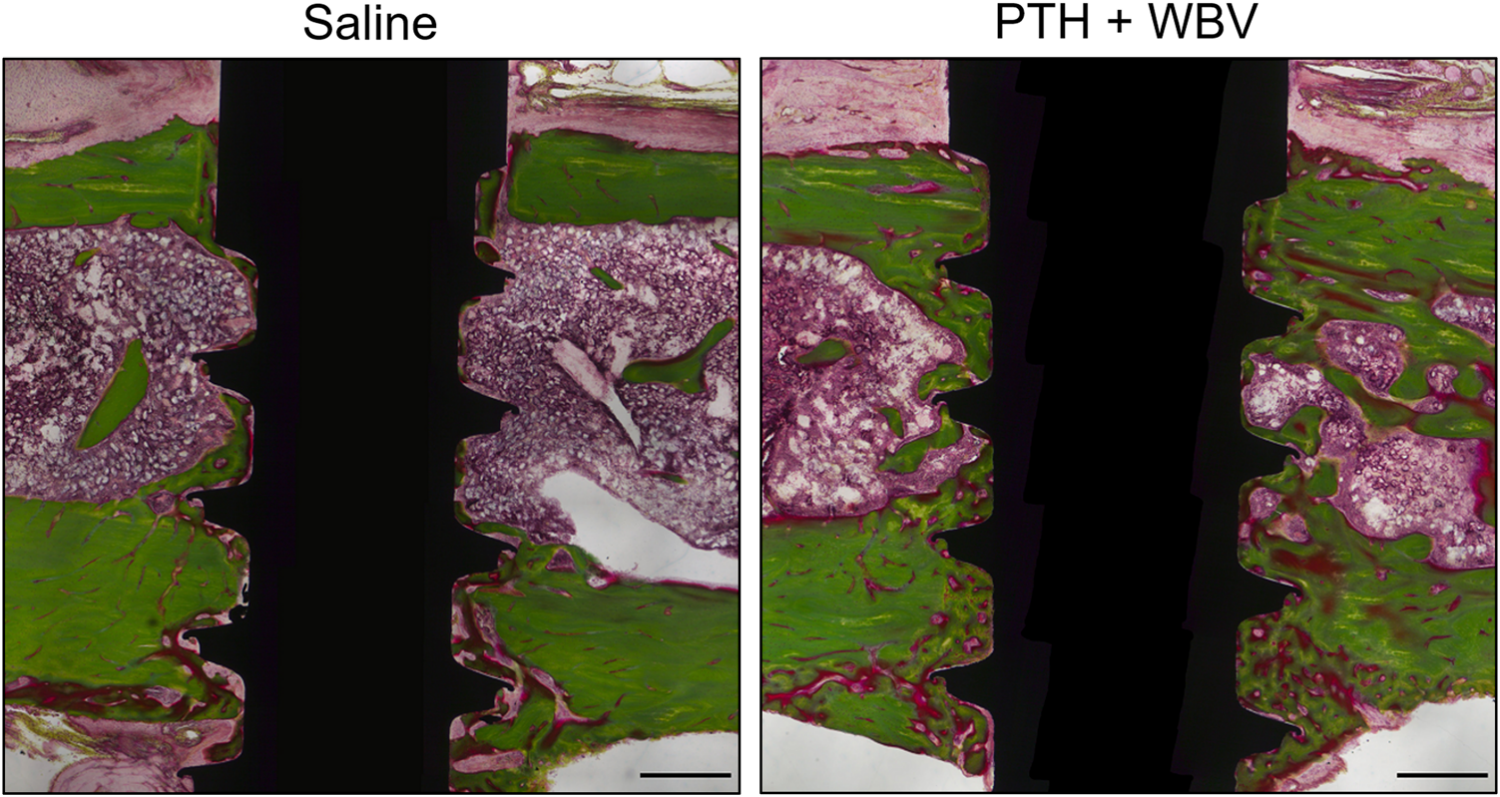
Representative histological sections stained with Villanueva–Goldner. Histologic sections compared with the test (PTH+WBV) and control (saline) groups after 4 weeks of healing. Scale bar: 500 μm. PTH, parathyroid hormone; WBV, whole-body vibration

Figure 3 shows representative images of the PTH + WBV and saline groups after 4 weeks of healing. In peri-implant trabecular bone of the drug-treated groups, specifically the PTH-treated groups, bone neoformation was observed. There was little mature bone around the implants in the saline group. In peri-implant cortical bone, the bone width seemed to have thickened by the loading and drug treatments.

**Fig. 4:**
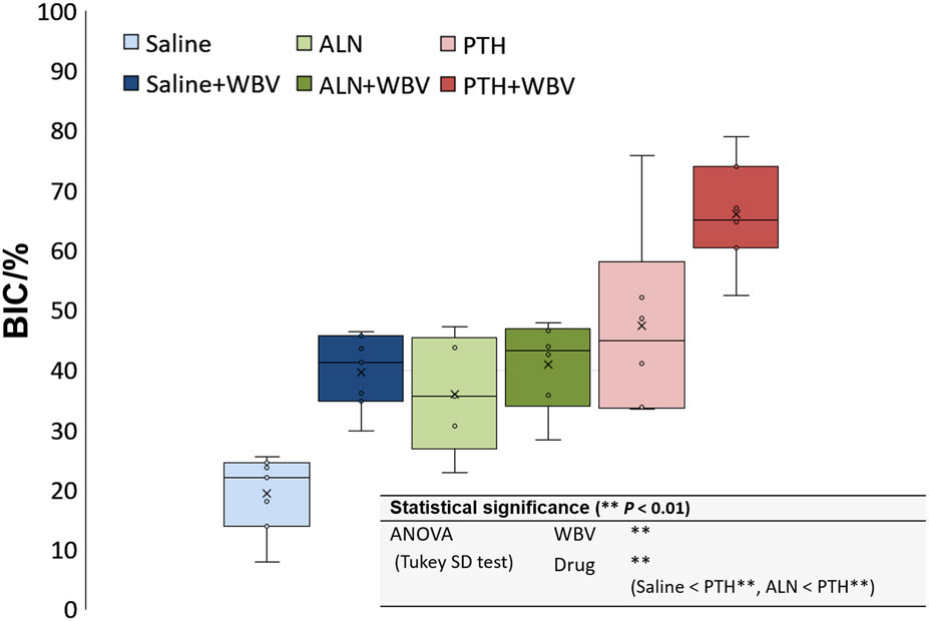
Histomorphometric results of bone-to-implant contact (BIC). The box-plot of BIC (**P < 0.01; two-way ANOVA followed by Tukey’s HSD test). PTH, parathyroid hormone; WBV, whole-body vibration; ALN, alendronate

**Fig. 5:**
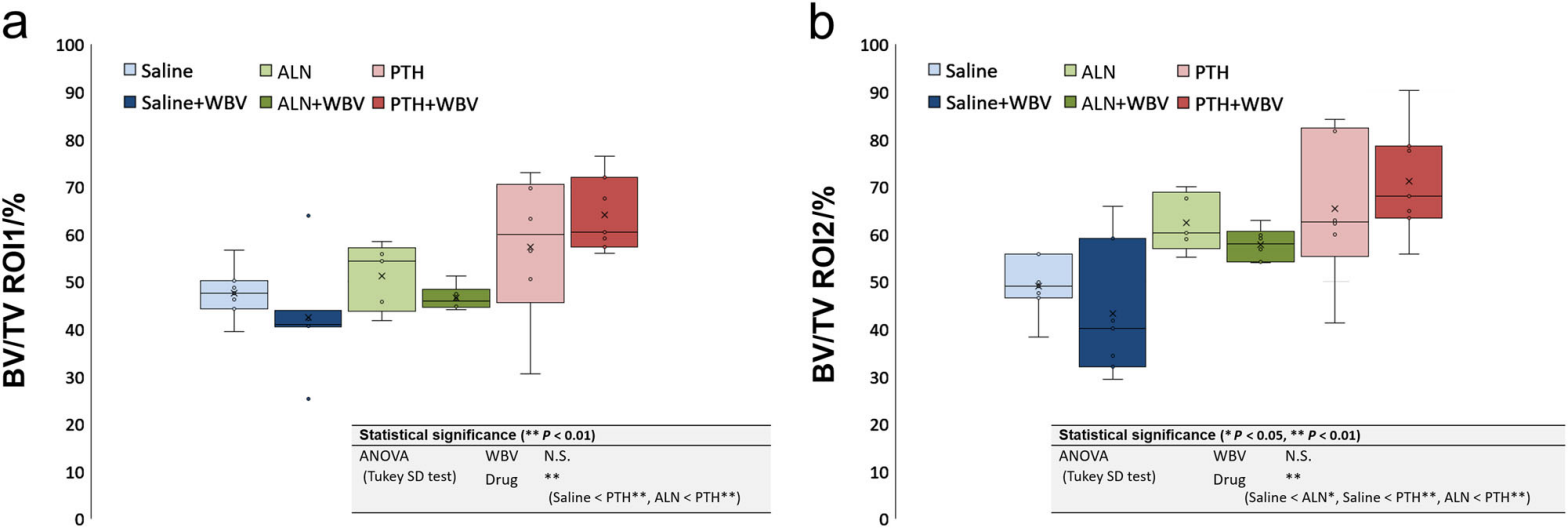
Histomorphometric results of the bone volume (BV)/tissue volume (TV). The box-plot of the BV/TV of each region of interest (**a** ROI1 and **b** ROI2) (*P < 0.05, **P < 0.01; two-way ANOVA followed by Tukey’s HSD test). PTH, parathyroid hormone; WBV, whole-body vibration; ALN, alendronate

In the histomorphometric analysis, BIC was significantly increased by the loading and the PTH administration (*P* < 0.01) (Fig. 4). BV/TV ROI1 and ROI2 were significantly enhanced by the PTH administration (*P* < 0.01) (Fig. 5a, b).

## Discussion

This study investigated the osteogenic impact of LMHF loading and anti-osteoporosis medications on peri-implant bone healing and implant osseointegration in an osteoporosis model, and assessed their combined effects on these processes. The main findings of this study are as follows: (i) LMHF loading and PTH have additive effects on peri-implant bone healing and implant osseointegration in osteoporosis model. (ii) PTH administration is more effective than BP administration on preventing the OVX-induced impaired peri-implant bone response. (iii) LMHF loading and drug administration act locally on the bone healing process.

The assessed bone formation parameters in the PTH + WBV group tended to be highest after 4 weeks of healing, indicating that PTH and WBV additively facilitate implant osseointegration. Interestingly, no obvious positive effect was found in the ALN + WBV groups. Similarly, a comparable study reported that the combination of ALN and LMHF loading did not lead to an additive reaction influencing the bone healing response.^21^ This might be because ALN inhibits osteoclastic bone activity, which is required in the process of bone adaptation and, therefore, implant osseointegration. Collectively, these results indicate that LMHF loading and PTH administration additively increase the osteogenic response in peri-implant bone, achieving early bone healing and strengthening implant osseointegration, and that PTH combined with LMHF loading has a bone-stimulating effect superior to that of ALN and LMHF loading.

Regarding anti-osteoporosis medications, the overall results of the post hoc analysis (Tukey’s HSD test) clearly showed that PTH was the medication that exhibited the most pronounced bone-stimulating effect compared with ALN and control (saline), based on RT value, BIC, and BV/TV. This affirmed that PTH administration has potent osteogenic capability to stimulate implant osseointegration. Almagro et al.^33^ reported that PTH administration improves implant osseointegration by increasing BIC and peri-implant bone mineral density in a rabbit model of ovariectomy and glucocorticoid-induced osteoporosis, findings that align with the present study. A clinical feasibility study also described the tendency for new bone formation around mini implants obtained from individuals on short-term treatment with teriparatide.^32^ Furthermore, we observed that ALN did not have a positive effect on peri-implant bone formation compared with PTH. These results suggest that such a promotional effect on new bone formation depends on osteoblast activity around implants.^39^ However, some previous studies reported that BP stimulates implant osseointegration in comparable animal models,^35^ which might be because of using the different osteoporosis animal model or dosing regimen of BP administration. There are still many controversial aspects regarding this point. Actually, the influence of BP administration on implant success has not been completely determined in clinical practice.^40–44^

Remarkably, micro-CT analysis revealed differences in effect profiles between LMHF loading and PTH. This means that the RG value of cortical bone was increased by the loading. In contrast, the RG value of trabecular bone was increased by the PTH administration. These results suggest that they act locally on the bone healing process. Zhang et al.^17^ reported that only the cortical BIC significantly increased with high-frequency loading for 4 weeks compared with the unloaded control, suggesting that LMHF loading increased the degree of contact between cortical bone and the implant. Shirota et al.^31^ reported that bone volume density and BIC around implants in the OVX group treated with PTH was almost the same as that of the sham-operated group throughout the observation period. The researchers suggested that intermittent PTH administration not only can prevent resorption of newly generated trabeculae around implants but also can recover bone volume lost because of ovariectomy.

This study has some limitations. The study was planned as a basic study to examine the fundamental phenomenon in osteoporotic models, before progressing to an oral implant model using higher-level experimental animals. As long bones such as the tibia are different from craniofacial bones, further studies using jawbone models are necessary to optimize the performance of vibration devices for local application and explore the optimal timing and duration of PTH administration for peri-implant bone healing and implant osseointegration in osteoporosis. However, the rat tibia, which has been used successfully in previous experiments,^15–18^ is considered a suitable and reliable location for implant surgery and can be maintained without unpredictable loading. Therefore, the findings of the current study will increase our understanding of the peri-implant bone response to loading and drugs, prior to subsequent studies using an oral implant model.

Osteoporosis could potentially complicate oral implant treatment because of disease-specific characteristics such as abnormal bone conditions and poor bone healing ability. These problems must be resolved by proper management of osteoporotic condition to ensure that oral implant treatment is successful in osteoporotic patients. In summary, the findings of present study suggest that both LMHF loading and intermittent PTH administration have osteogenic potency on peri-implant bone. They also suggest that they act locally and additively on the bone healing process, improving the condition of implant osseointegration. This could be a new therapeutic option for oral implant treatment in osteoporotic patients, avoiding problems such as delayed bone healing and failure of osseointegration.

## Materials and methods

### Animals

Forty-four female Wistar rats (age, 11 weeks; average weight, (171.9 ± 8.7) g) were used in this study. The rats were kept under climate-controlled conditions (23.5 °C, 50% humidity, 12-h light/dark cycle) and had free access to standard laboratory diet and tap water.

The study was approved by the Institutional Animal Care and Use Committee of the Tohoku University Environmental & Safety Committee, and was carried out at the Institute for Animal Experimentation at Tohoku University Graduate School of Medicine.

### Experimental design

**Fig. 6:**
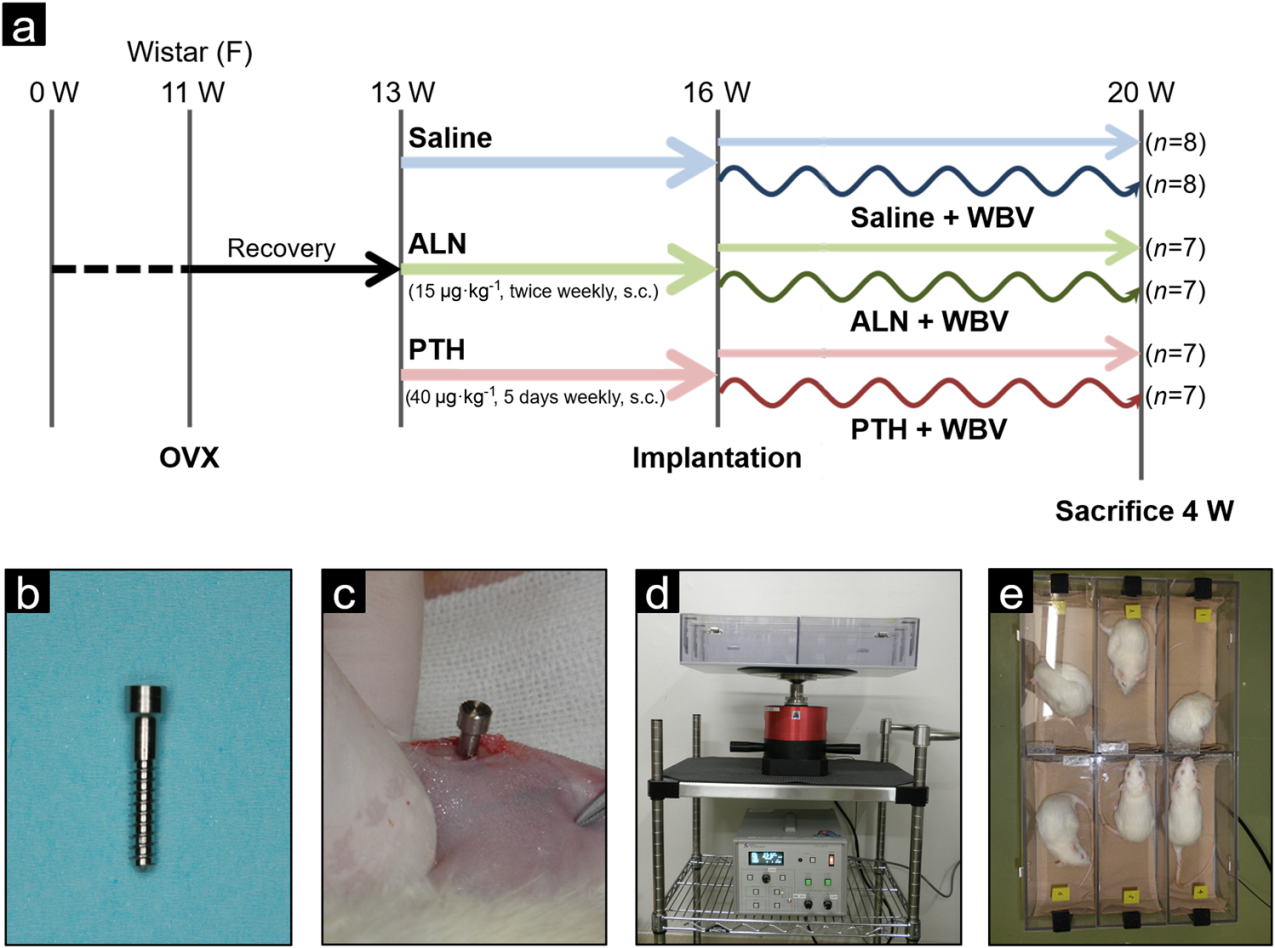
Experimental protocol and devices. **a** Illustration of the experimental design and grouping. **b** Custom-made titanium implants (Ø: 2 × 13 mm^2^). **c** The implant was inserted into the proximal metaphysis of the tibiae, perpendicular to the long axis of the tibia. **d** Vibration device. **e** Animal box set onto the device. Six rats could simultaneously undergo vibration loading

Figure 6a illustrates the experimental design. At 11-weeks-old rats were ovariectomized to induce osteoporosis. Two weeks after ovariectomy, rats were divided into six groups: saline (*n* = 8), saline + WBV (*n* = 8), alendronate (ALN) (*n* = 7), ALN + WBV (*n* = 7), PTH (*n* = 7), and PTH + WBV (*n* = 7). The ALN-treated groups (ALN and ALN + WBV) were injected subcutaneously twice per week with 15 μg·kg^-1^ of ALN (Wako Pure Chemical Industries, Osaka, Japan). The PTH-treated groups (PTH and PTH + WBV) were injected subcutaneously 5 days per week with 40 μg·kg^-1^ of PTH (hPTH(1-34); Peptide Institute, Osaka, Japan). Rats in the saline and saline + WBV groups were administered volume-matched subcutaneous injections of saline. Administration of drugs was continued until the rats were killed. The period from ovariectomy to the initiation of medication was set so that rats had reached the early stages of osteoporosis with the resultant abnormal conditions regulating bone metabolism.^45^ The dosage of ALN was determined by reference to previous studies,^46,47^ and was based on preclinical studies in OVX rats that demonstrated a significant increase in bone mass and strength.^48^ The dosage is comparable with the 20 mg per day dosage prescribed to treat osteoporosis on a mg·kg^-1^ basis. The dosage of PTH was determined by reference to a previous study,^31^ and lies within the dose range investigated in a dose-dependency study.^49^ These relatively high dosages were chosen to maximize the possibility of bone–implant osseointegration becoming established.

Three weeks following the onset of drug administration (5 weeks post ovariectomy), a custom-made titanium implant (2 × 13 mm^2^; cp-Titanium Grade 2, machine surface) was inserted into the proximal metaphysis of both tibiae in rats in all six groups (Fig. 6b, c). Rats were anaesthetized with 2.5% isoflurane (Escain; Mylan, Pittsburgh, PA, USA). Both cortices of the tibia were perforated at a low rotational speed under constant saline cooling with a surgical drill, which was 0.2 mm smaller than the implant’s diameter to achieve good primary stability. The implants were inserted by means of a custom-fit wrench with manual torque. After implant insertion, wounds were closed using 4-0 polyglycolic acid sutures (Matsuda Ika Kogyo, Tokyo, Japan).

In the three groups of rats that underwent WBV (saline + WBV, ALN + WBV, and PTH + WBV), LMHF loading was applied starting the day after implant installation via WBV at a frequency of 50 Hz and a magnitude of 0.5 *g* for 15 min per day, 5 days per week. Big Wave G-Master (Asahi Seisakusyo, Tokyo, Japan) was used as the vibration device (Fig. 6d, e).

Rats were killed 4 weeks following implantation by cervical displacement under isoflurane-induced anesthesia. The tibiae with the implants were then dissected.

### Evaluation of implant osseointegration

*Removal torque test*: One implant per rat was used to perform the removal torque test. The tibiae were fixated with a jig that was perpendicular to the axis of the implant. A torque gauge (ATG1.5CN/ATG12CN; Tohnichi Mfg, Tokyo, Japan) was attached to the implant head. The peak loosening torque (i.e., the removal torque (RT) value) was recorded.

**Fig. 7:**
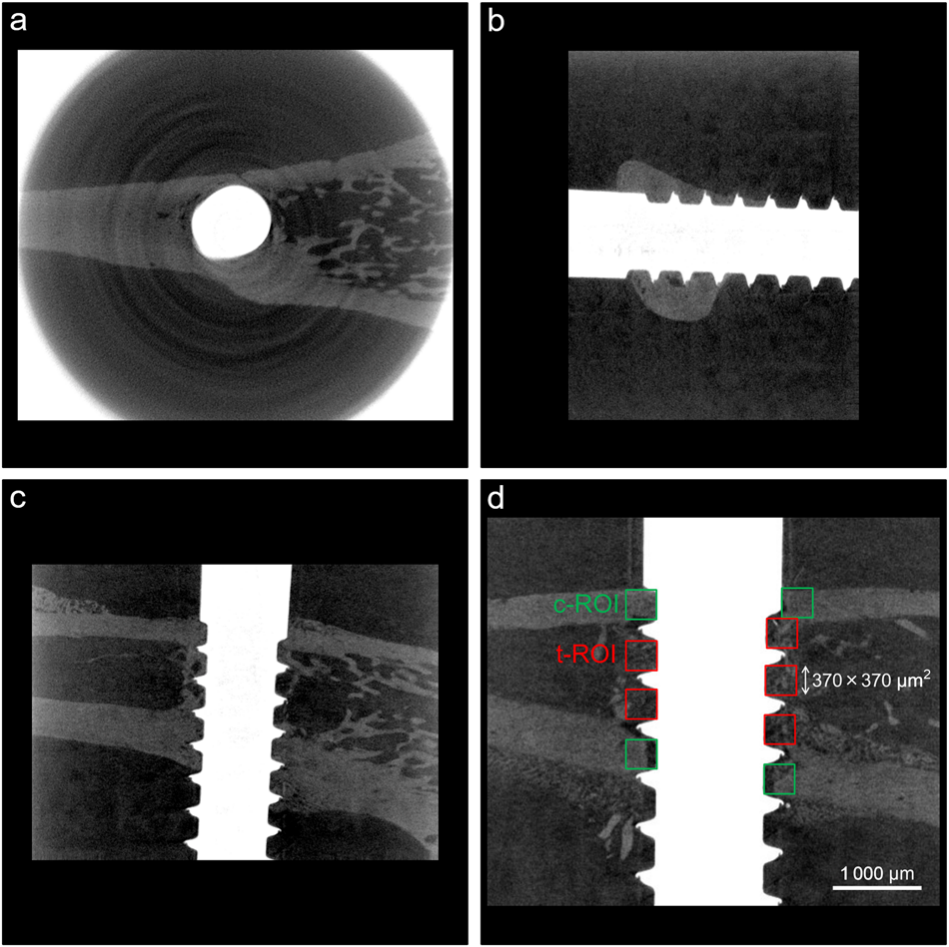
Evaluation methods of micro-CT analysis. **a** Axial, **b** coronal, and **c** sagittal images of three-dimensional multiplanar reconstruction (3D MPR). **d** Illustration of the region of interest (ROI) for micro-CT analysis. (c-ROI and t-ROI: 0.37 × 0.37 mm^2^ (26-pixel) square in the cortical and trabecular bone adjacent to the implant surface)

*Micro-CT analysis*: Another implant per rat was used for micro-CT analysis. Each sample, positioned in a water-filled plastic cylinder (29 × 57 mm^2^), was scanned using a micro-CT system (ScanXmate-D225RSS270; Comscantecno, Kanagawa, Japan), which was set to 200 kV and 100 μA. Three-dimensional sectional images were reconstructed with 928 × 736 pixel resolution, in which the pixel size was 0.254 mm and the isotropic voxel size was 10.68 μm (Fig. 7a–c). From each 3D multiplanar reconstruction (MPR) images data set, a sagittal slice along the axis of the tibia and implant was selected at the center of the implant as 32-bit grayscale image data. A 32-bit gray value from 0 to 255 was measured using ImageJ software (U.S. National Institutes of Health, Bethesda, MA, USA; http://imagej.nih.gov/ij/, 1997–2014). The region of interest (ROI) was set as a 0.37 × 0.37 mm^2^ (26-pixel) square in the cortical and trabecular bone adjacent to the implant surface (c-ROI and t-ROI). A relative gray (RG) value of the cortical and trabecular bone was calculated whereby the gray value of the water and implant was defined as the reference value (0 and 100, respectively) (Fig. 7d).

*Histologic and histomorphometric analyses*: After micro-CT analysis, the bone–implant blocks were fixed in phosphate-buffered formalin solution and dehydrated in a series of increasing concentrations of alcohol. After dehydration, samples were embedded in poly(methyl methacrylate). Embedded samples were cut using a diamond saw (Exakt BS-300CP; Exakt Technologies, Norderstedt, Germany) along the axis of the tibia and implant. After polishing to a final sample thickness of 40 μm (Exakt MG-400CS; Exakt Technologies), sections were stained with Villanueva–Goldner stain.

**Fig. 8:**
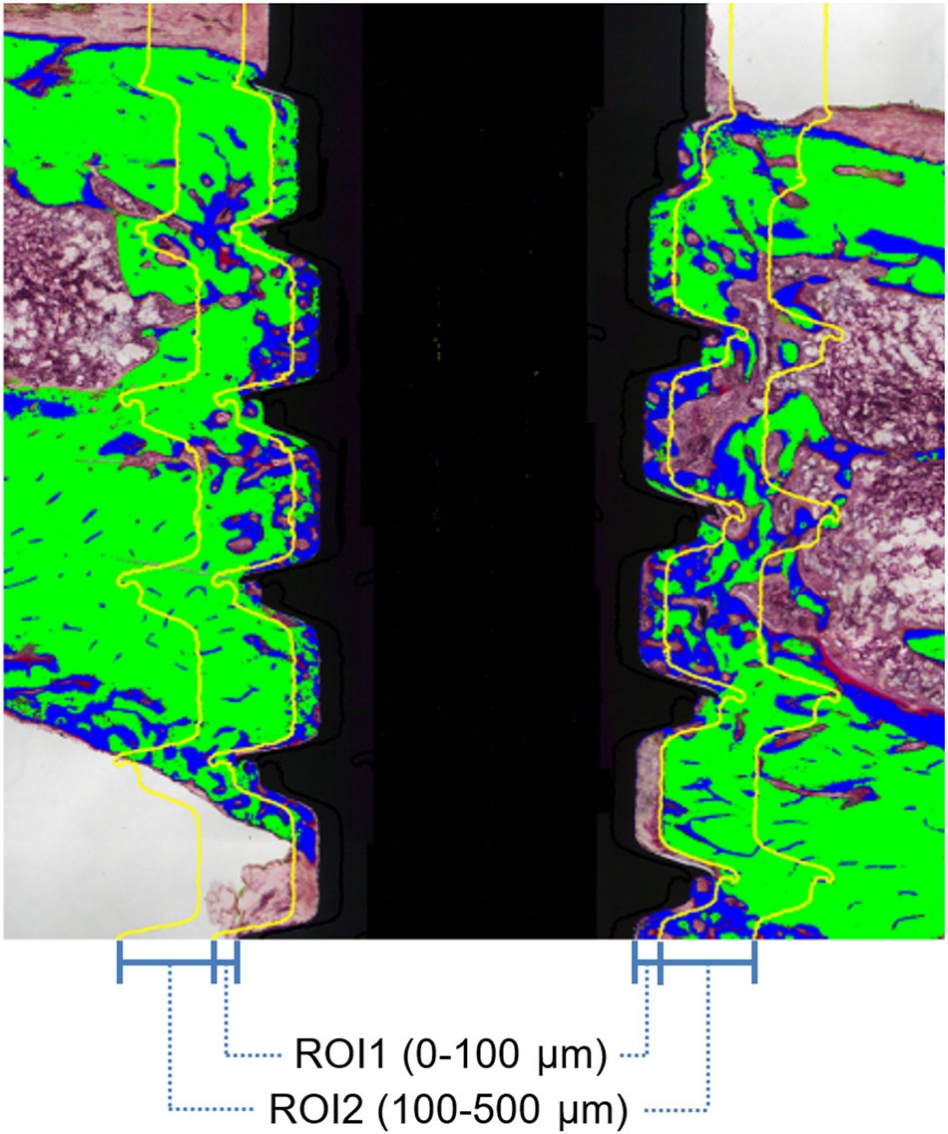
Evaluation methods of histomorphometric analysis. Illustration of the reference sites (ROI1: 0 (implant surface)–100 μm, ROI2: 100–500 μm) for the bone volume (BV)/tissue volume (TV) evaluation

Histologic and histomorphometric analyses were performed using a light microscope at a magnification of ×100 (Leica DM3000; Leica Laborlux, Wetzlar, Germany). Samples were scanned with a high-sensitivity camera (Leica DFC295; Leica Laborlux) at an image resolution of 1.28 μm per pixel. Histomorphometric analysis was performed via digital imaging processing (Adobe Photoshop CS6; Adobe Systems, San Jose, CA, USA and ImageJ, U.S. National Institutes of Health). The following analyses were performed: (i) Bone-to-implant contact (BIC; %): (summation of the length of contact between the bone and implant (μm)/implant length extending from the most medial to most lateral BIC point (μm)) × 100; and (ii) Peri-implant bone volume relative to tissue volume (BV/TV; %): (area occupied by bone (μm^2^)/reference area (μm^2^)) × 100. Two regions of interest (ROI) were defined: 0–100 μm (BV/TV ROI1) and 100–500 μm (BV/TV ROI2) zones extending from the implant surface. The areas encompassed the peri-implant tissues from the medial to lateral cortex (Fig. 8). BIC and BV/TV measurements were performed on both the proximal and distal implant sides.

### Statistical analysis

Statistical analyses were conducted using SPSS (ver. 13.0; SPSS Inc., Chicago, IL, USA). Two-way analysis of variance was performed to evaluate differences among the drug administration (ALN, PTH, or saline) and the loading (with or without WBV). The Tukey’s honest significant difference (HSD) test was performed to compare differences between groups. A *P*-value < 0.05 was considered statistically significant.
